# Mean cerebral blood volume is an effective diagnostic index of recurrent and radiation injury in glioma patients: A meta-analysis of diagnostic test

**DOI:** 10.18632/oncotarget.14922

**Published:** 2017-01-31

**Authors:** Zhanzhan Li, Qin Zhou, Yanyan Li, Shipeng Yan, Jun Fu, Xinqiong Huang, Liangfang Shen

**Affiliations:** ^1^ Department of Oncology, Xiangya Hospital, Central South University, Changsha, Hunan Province 410008, China; ^2^ Office of Cancer Prevent and Control, Xiangya Hospital, Central South University, Changsha, Hunan Province 410008, China; ^3^ Office of Cancer Prevent and Control, Hunan Provincial Tumor Hospital and The Affiliated Cancer Hospital of Xiangya School of Medicine, Central South University, Changsha, 410013 China

**Keywords:** glioma, mean cerebral blood volume, early diagnosis, meta-analysis

## Abstract

We conducted a meta-analysis to evaluate the diagnostic values of mean cerebral blood volume for recurrent and radiation injury in glioma patients. We performed systematic electronic searches for eligible study up to August 8, 2016. Bivariate mixed effects models were used to estimate the combined sensitivity, specificity, positive likelihood ratios, negative likelihood ratios, diagnostic odds ratios and their 95% confidence intervals (CIs). Fifteen studies with a total number of 576 participants were enrolled. The pooled sensitivity and specificity of diagnostic were 0.88 (95%CI: 0.82-0.92) and 0.85 (95%CI: 0.68-0.93). The pooled positive likelihood ratio is 5.73 (95%CI: 2.56-12.81), negative likelihood ratio is 0.15 (95%CI: 0.10-0.22), and the diagnostic odds ratio is 39.34 (95%CI:13.96-110.84). The summary receiver operator characteristic is 0.91 (95%CI: 0.88-0.93). However, the Deek's plot suggested publication bias may exist (t=2.30, P=0.039). Mean cerebral blood volume measurement methods seems to be very sensitive and highly specific to differentiate recurrent and radiation injury in glioma patients. The results should be interpreted with caution because of the potential bias.

## INTRODUCTION

Gliomas represent approximately 30% of all central nervous system tumors and 80% of malignant brain tumors [1]. The methods of 6-week radiation therapy and concomitant temozolomide chemotherapy and 6 times of adjuvant temozolomide chemotherapy after surgical resection are widely employed in the treatment scheme [2]. However, this treatment protocol increased the risk of brain tissue radiation injury and recurrent in patients with glioma. One of the most common treatment-related symptoms is pseudoprogression. It is defined that growth of existing lesions or appearance of new lesions within 12 weeks of completion of radiation therapy may be the result of treatment effects rather than growth of tumor. During continued follow-up, if lesion stabilizes or shrinks, the initial growth is confirmed pseudoprogression [3]. Previous studies also suggested that enlarged and enhanced lesions on MR images may represent pseudoprogression in 46.8-64% of the cases [4]. Much effort has been taken to distinguish the progression from pseudoprogression through advanced MR imaging techniques, such as DWI and dynamic susceptibility contrast PWI. Because the ADC values in necrotic are usually higher than recurrent tissue. However, this method is limited because of the differences of tumor type. Reduced diffusion can represent highly cellular tumor areas and inflammatory [5].

The ways of dealing with the radiation injury and recurrent of tumor are largely different in clinical practice. It is important to differentiate the radiation injury and recurrent. The perfusion-weighted imaging technical is an advanced imaging method, and has been widely used in the diagnostic of tumor. The cerebral blood volume (rCBV) is one of the perfusion-weighted imaging index, and can evaluate the blood supply and density of microvessel. Kim et al reported a histogram analysis of high relative cerebral blood volume, the sensitivity and specificity are 90.2% and 91.1% with a cut-off of 1.7 for differentiating tumor recurrence and radiation injury s [6]. However, CBV also has some limitations because most of lesions have variable tumor fractions. The CBV from tumoral comments may affect cerebral blood volume itself. To overcome the shortage of these methods, Cha et al compared the subtracted histogram mode with a multiparametric approach, and found that the latter method is more accurate, with 81.8% sensitivity and 100% specificity [7]. Martinez et al reported that rCBV was useful for differentiating between pseudoprogression and true progression in our sample, with sensitivity=100% and specificity=100% for rCBV [8]. Some studies have reported the diagnostic value of rCBV mean in recurrent and radiation injury. However, all of them were limited in some factors such as small sample size. The aim of this study was to give an accurate and systematical evaluation for diagnostic value of recurrent and radiation injury in glioma patients.

## RESULTS

### Study selection and study characteristics

**Figure 1:**
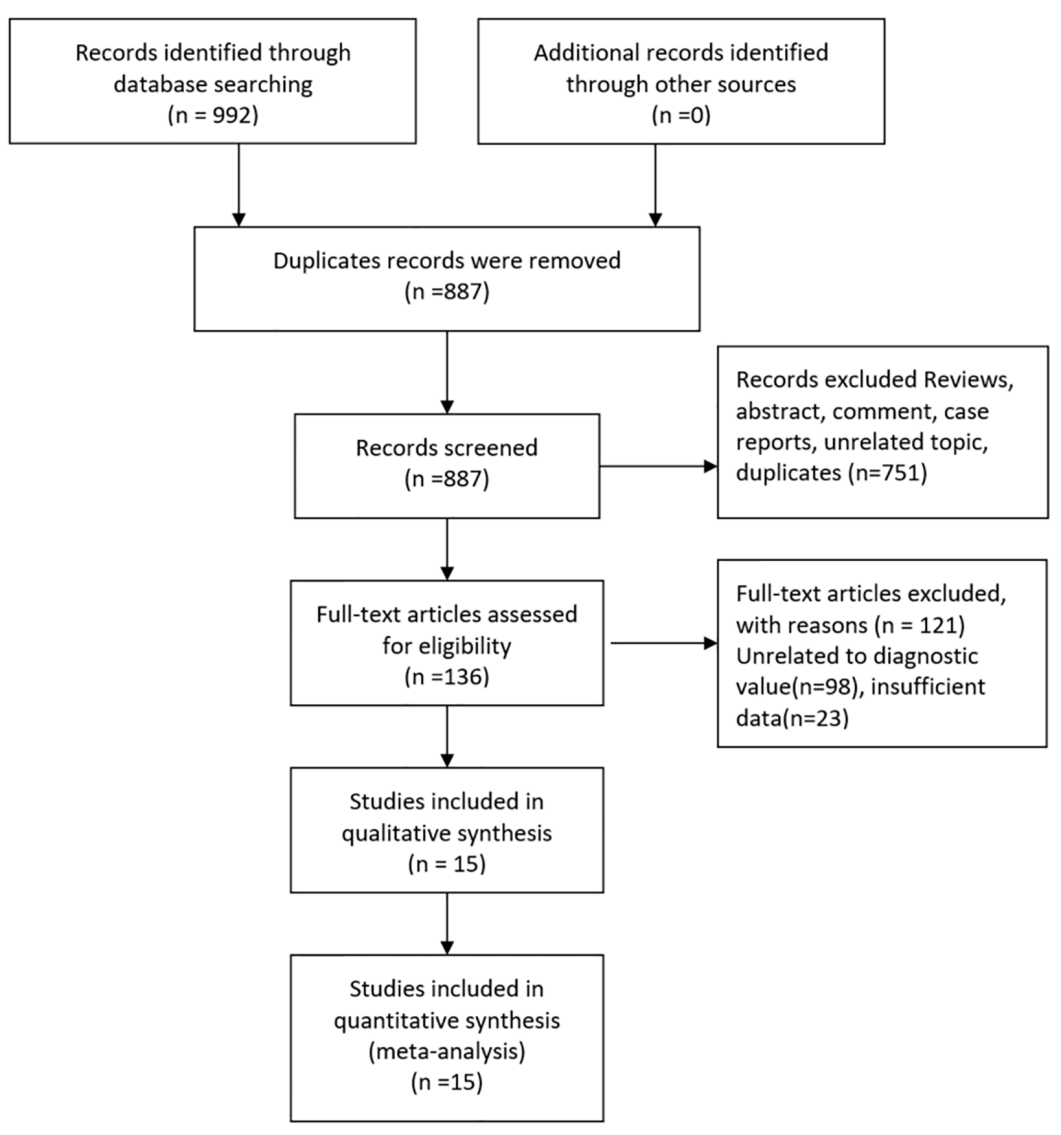
Flow diagram of studies selection process

The initial search returned 992 records, of which 105 were excluded for duplicate studies. Then 872 studies were excluded for various reasons (comments, reviews, case reports, or not relevant studies) on the basis of title, abstract and full text. The remaining 15 records were included in the final analysis [6–8, 9–20]. Please see more details in Figure 1.

The main characteristics of the included studies are presented in Table 1. The included studies were published from 2009 to 2015. In total, 576 participants with glioma are enrolled. The reference standard of include studies is from clinical or history pathology. Chemotherapy patients received are Temozolomide, and some of them also received radiation treatment. Gender ratio among studies is comparable.

**Table 1:** Characteristics of the included studies in the meta-analysis

Author	Year	Country	Meanage(y)	Gender (M/F)	SampleSize	Radiation doses(Gy)	Chemotherapy	Cut-off	TP	FP	FN	TN
Barajas	2009	USA	54.2	33/24	57	59.4	Tenozolomide	1.75	36	6	10	14
Hu	2009	USA	47.7	11/2	40	63	-	0.71	22	0	2	16
Bobek	2010	Poland	38.5	3/5	11	60	Tenozolomide	1.25	3	0	2	6
Kim	2010	Korea	46.1	8/2	10	60.1	Tenozolomide	3.69	4	0	0	6
Ozsunar	2010	Turkey	42.0	22/8	32	-	-	1.30	19	3	3	7
Prat	2010	Spain	-	14/10	24	-	-	1.0	8	1	0	9
Hu	2011	USA	-	-	31	-	-	1.14	13	2	2	14
Seeger	2013	Germany	53.6	24/16	40	60	Tenozolomide	2.25	19	4	4	13
Wang	2013	China	47.0	15/8	23	>54	-	1.3	9	1	4	9
Young	2013	USA	58.0	14/6	20	59.4	Tenozolomide	2.4	16	1	0	3
Cha	2014	Korea	49.0	18/17	35	-	Tenozolomide	1.8	9	4	2	20
Di	2014	Italy	62.5	18/11	29	60	Tenozolomide	NA	18	1	3	7
Martinez	2014	Spanish	48.0	14/20	34	57.7	Tenozolomide	0.9	17	0	0	17
He	2014	China	45.0	60/34	94	-	-	NA	53	25	7	9
Yin	2015	China	43.0	50/46	96	-	-	NA	67	18	5	6

### Quality evaluation

The evaluation of quality is shown in Supplementary Figure 1 and Supplementary Figure 2. As we can see, 3 studies in patient's selection, 1 study of index test, 3 study of reference standard, and 1 of flow and timing are considered as high risk bias. For applicability concerns, 3 studies of index test and 3 studies of reference standard are treated as high concern. But viewed as a whole, the quality of included studies is relevant high.

### Pooled diagnostic values

**Figure 2:**
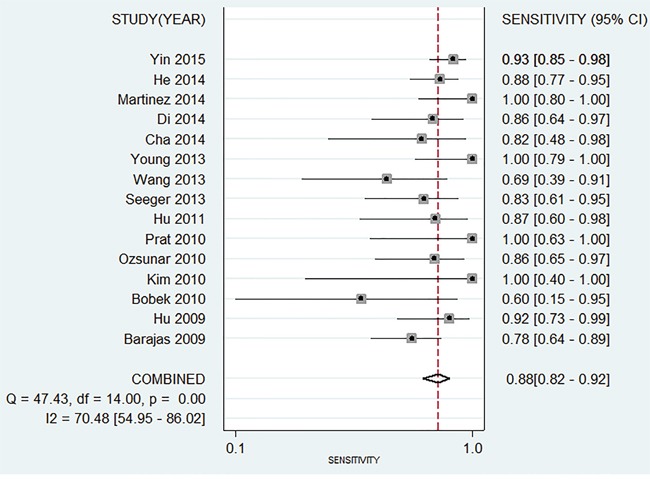
Forest plot of pooled sensitivity of mean cerebral blood volume for recurrent and radiation injury in glioma patients

**Figure 3:**
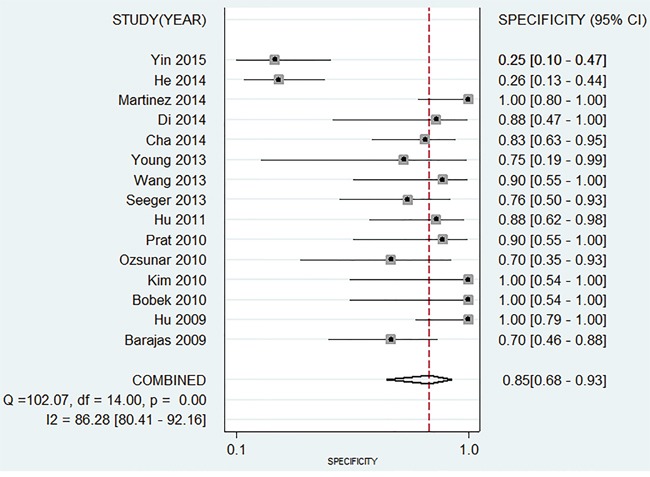
Forest plot of pooled specificity of mean cerebral blood volume for recurrent and radiation injury in glioma patients

**Figure 4:**
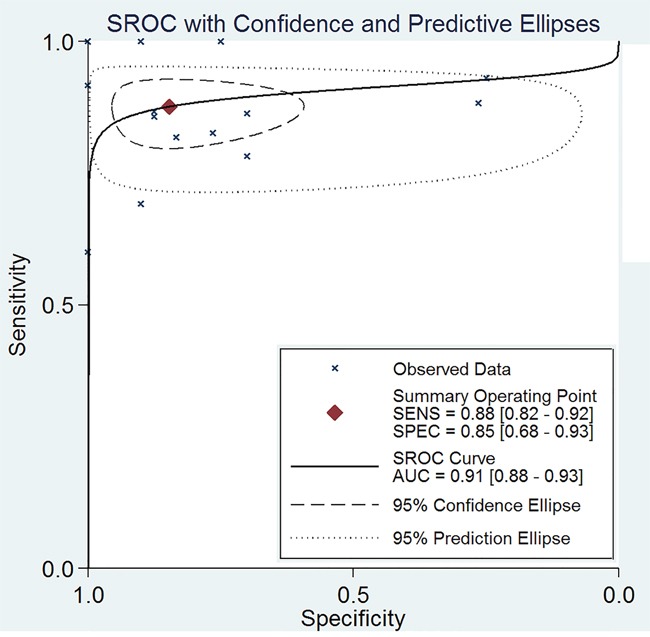
The SROC curve of mean cerebral blood volume for recurrent and radiation injury in glioma patients

**Figure 5:**
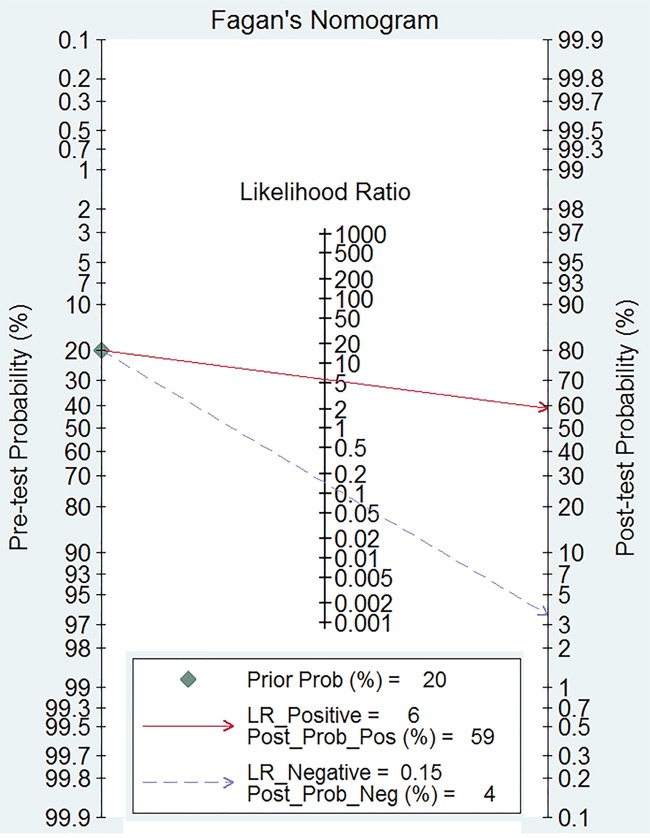
Fagan diagram evaluating the overall diagnostic value of mean cerebral blood volume for recurrent and radiation injury in glioma patients

The Spearman test indicated that there is no threshold effect within studies (r=-0.099, *P*=0.741). Random-effects models were used in present analyses because the heterogeneity within studies are more than 50%. The pooled sensitivity and specificity were 0.88 (95%CI: 0.82-0.92, Figure 2) and 0.85 (95%CI: 0.68-0.93, Figure 3). The pooled positive likelihood ratio is 5.73 (95%CI: 2.56-12.81), negative likelihood ratio is 0.15 (95%CI: 0.10-0.22), and the diagnostic odds ratio is 39.34 (95%CI: 13.96-110.84). The summary receiver operator characteristic is 0.91 (95%CI: 0.88-0.93, Figure 4). The diagnostic accuracy of mean cerebral blood volume is relatively high. The Fangan plot was shown in Figure 5. If the pre-test probability is 20%, the post-test probability is approximately 59% through positive likelihood ratio and 4% through negative likelihood ratio. The diagnostic accuracy is high.

### Sensitivity analysis and publication bias

**Figure 6:**
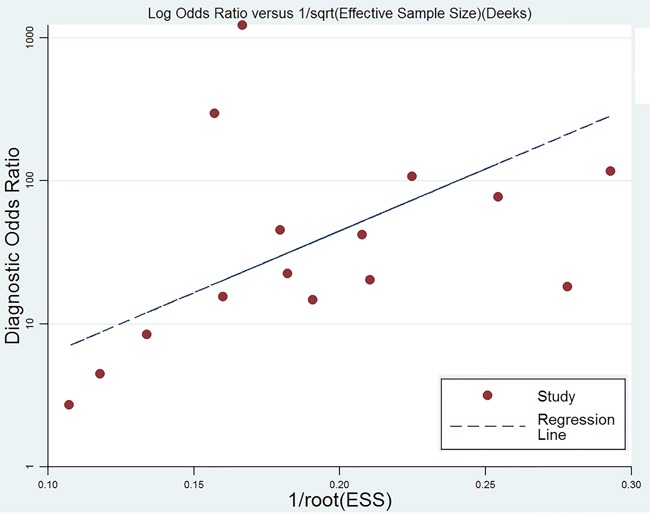
Deek's funnel plot to evaluate the publication bias

We conducted sensitivity analyses through sequentially excluding some certain studies, and the summary sensitivity and specificity, positive likelihood ratio, negative likelihood ratio and summary receiver operator characteristic were altered (data were not given), indicating that the pooled estimations were stable. We used Deek's plot to evaluate the publication bias. The bias test shown existence of publication bias (t=2.30 P=0.039) as indicated in Figure 6.

## DISCUSSION

Our study suggested that mean cerebral blood volume could aid in the prediction of the presence of recurrent and radiation injury in glioma patients (AUC=0.91). The mean cerebral blood volume showed high sensitivity (0.88, 95%CI: 0.82-0.92) and specificity (0.85, 95%CI: 0.68-0.93). These data indicate that mean cerebral blood volume is a useful diagnostic tool for differentiate recurrent and radiation injury in glioma patients.

The perfusion-weighted imaging is mainly used to evaluate the vessel physiological status and hemodynamic [21, 22]. Many parameters of perfusion are involved in the progression and prognosis of tumor. The rapid growth and proliferation in glioma needs nutrients and oxygen. The lack of supply will stimulate the tumor cell to secret many vascular growth factors and accelerate the tumor angiogenesis. Blood perfusion and volume from tumor tissue will also increase. However, this kind of tumor vessel is different from normal one. The hemodynamics in the brain tissue will change, especially in tumor lesions. The necrosis areas of cerebral tissue caused by radiation therapy presented different characteristics. The radiation can lead to a series of tissue injury, such as vascular endothelial cell injury, hemal wall thinning, transparent degeneration and fibrin necrosis, resulting in vascular occlusion and brain tissue necrosis [23]. The blood perfusion of necrosis areas caused by radiation will be reduced. The clinical parameters of perfusion-weighted imaging could differentiate the recurrent and necrosis lesions from the pseudoprogression. Our results found mean cerebral blood volume is an effective tool for recurrent and radiation injury in patients with glioma. Higher diagnostic odds ratio means better diagnostic values (0-100) The diagnostic odds ratio is 39.4. The positive likelihood ratio and negative likelihood ratio can explain the diagnostic value better. The two parameters are 5.73 and 0.15, which means probability of glioma recurrence is higher than radiation injury by 5.73 times when mean cerebral blood volume of lesions is higher than cut-off value. The probability is 15% when lesions area is lower that cut-off value. All of these showed the diagnostic accuracy of mean cerebral blood volume in patients with glioma.

The present meta-analysis has several strengths. First, a series of study focused on the diagnostic value of mean cerebral blood volume for recurrent and radiation injury in patients with glioma. As far as we know, this is the first meta-analysis and systematic review to the evaluate the diagnostic value of mean cerebral blood volume for glioma patients. Second, our meta-analysis is strictly in accordance with the latest Preferred Reporting Items for Systematic Reviews and Meta-Analyses Protocols. Also, most of all included studies were relevant high-quality. Third, study selection, data extraction, and evaluation of the risk of bias were carried out by two authors independently, which effectively reduces the risk of selection bias.

Several limitations of this meta-analysis merit consideration. The present meta-analysis showed significant heterogeneity across the included studies. The results of meta-analysis should be interpreted with cautions due to the heterogeneity. The threshold effects test showed no significance (r=0.099, P=0.741), which means other potential factors may influence the sources of heterogeneity. The reasons could be as following: First, the included studies in the meta-analysis did not use the same cut-off. Second, the clinical characteristics of study population, such as mean age, duration of disease, radiation doses, and chemotherapy among the selected studies. The available information showed that mean ages ranged from 38.5 to 62.5. Mean age and treatment methods are associated with recurrence and radiation injury of glioma. Thus, these factors could be a significant source of heterogeneity. Third, the population included in single study are from different areas, and the ethnicity may have a substantial effect on the diagnostic value [24]. Besides, we could not conduct subgroup analyses because the sample size of these is relevant small. Finally, the Deek's plot suggested the publication bias may exist, which potentially results in overstatement of the diagnostic value.

In conclusion, mean cerebral blood volume is an effective diagnostic tool for differentiating recurrent and radiation injury in glioma patient's glioma. It can effectively differentiate pseudoprogression from true progression in glioma patients. However, the results should be interpreted with caution because of the potential publication bias.

## MATERIALS AND METHODS

### Literature search

Ethical approval is not applicable for the present study since this is a meta-analysis based on previous published studies. The present meta-analysis is in accordance with the Preferred Reporting Items for Systematic Reviews and Meta-Analyses Protocols (PRISMA-2009 checklist) [25].

We identified relevant studies by searching PubMed, Web of Science, China National Knowledge Infrastructure, and Wanfang databases from inception to August 8, 2016. Electronic searches were performed using exploded medical subject heading and corresponding keywords, including “brain neoplasms OR glioma”, “perfusion MRI OR perfusion magnetic resonance imaging”. No language restriction was applicable. We also reviewed the reference lists of review articles to identify the other potentially eligible studies.

### Selection criteria

Two authors (LZZ and LYY) independently evaluated the eligibility of all studies. Any disagreements were resolved by fully discussion. The following criteria were required for included studies: (1) Study design: a retrospective or prospective study; (2) Patients: new or increased enhancing lesions appeared in the radiotherapy target area in patients with glioma; (3) Diagnostic: recurrent and radiation injury in glioma patients were diagnosed by perfusion-weighted Imaging; (4) Data: study could supply sufficient data for calculating four values (TP: true positives, FP: false positives, FN: false negatives, and TN: true negatives). Study focused on other intracranial tumors, that can't supply relevant data for analysis were excluded. When multiple publications were published from the same study, we used the one with the largest sample size.

### Data extraction

A standard Excel Sheet was used to collect data. LZZ conducted data extraction, and YSP checked the final data. The following information were extracted for included studies: surname of the first author, publication year, country, mean age of study population, gender ratio (Male/Female), sample size, reference standard, chemotherapy or radiation doses records, and values of screening (TP, FP, FN, TN). We tried to contact the authors of the study by e-mails for obtaining the relevant information if necessary.

### Quality evaluation

The Quality Assessment of Diagnostic Accuracy Studies 2 (QUADAS-2) was used to evaluate the quality of included studies [26]. The QUADAS-2 scale quantitated the quality of study through 4 key domains: patient selection, index test, reference standard, and flow of patients through the study and time of index tests and reference standard. Each domain consists of two section: bias of risk and applicability. If responses of all questions are YES, then we give a low risk bias judgment, or else potential bias risk may exist. Applicability are divided into three levels: high, unclear and low.

### Statistical analysis

We calculated the sensitivity, specificity, positive likelihood ratios (PLRs), negative likelihood ratios (NLRs), diagnostic odds ratios (DORs), summary receiver operator characteristic (SROC) curve area and their 95% confidence intervals (CIs) through bivariate mixed effects models [27]. Heterogeneity within studies were evaluated using Cochran Q statistic and quantified with I^2^ statistic. I^2^>50% and P<0.05 presented the existence of heterogeneity [28]. Fagan plots was used to show the relationship between the prior test probability, the likelihood ration, and posterior test probability, and Deek's funnel plot was used to evaluate the publication bias [29, 30]. We used the Stata 11.0 (Corp, College Station TX, USA) to conduct the whole analyses, and considered *P*<0.05 as a significant level.

## SUPPLEMENTARY MATERIALS FIGURES AND TABLES





## Abbreviations

rCBV: cerebral blood volume
TP: true positive
FP: false positive
FN: false negative
TN: true negative
QUADAS-2: Quality Assessment of Diagnostic Accuracy Studies
PLRs: positive likelihood ratios
NLRs: negative likelihood ratios
DORs: diagnostic odds ratios
SROC: summary receiver operator characteristic curve area
CI: confidence interval
