# Supplementary material for: Mean cerebral blood volume is an effective diagnostic index of recurrent and radiation injury in glioma patients: A meta-analysis of diagnostic test
Source: Oncotarget. 2017 Jan 31;8(9):15642–50. doi: 10.18632/oncotarget.14922 (PMC5362512; doi:10.18632/oncotarget.14922)
Supplement: Supplementary file 1 [file oncotarget-08-15642-s001.pdf]

Mean cerebral blood volume is an effective diagnostic index of recurrent and radiation injury in glioma patients: A meta-analysis of diagnostic test

SUPPLEMENTARY FIGURES AND TABLE

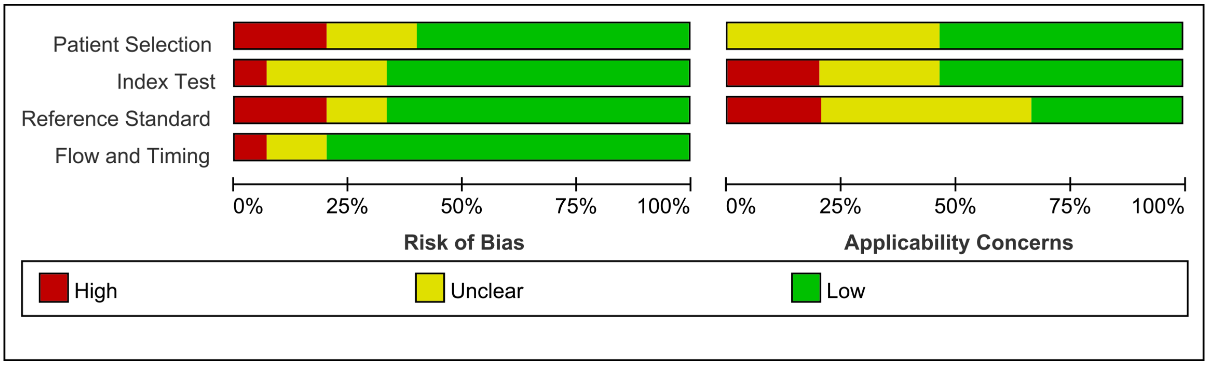

Supplementary Figure 1: Risk of bias and applicability concerns graph: review authors' judgements about each domain presented as percentages across included studies.

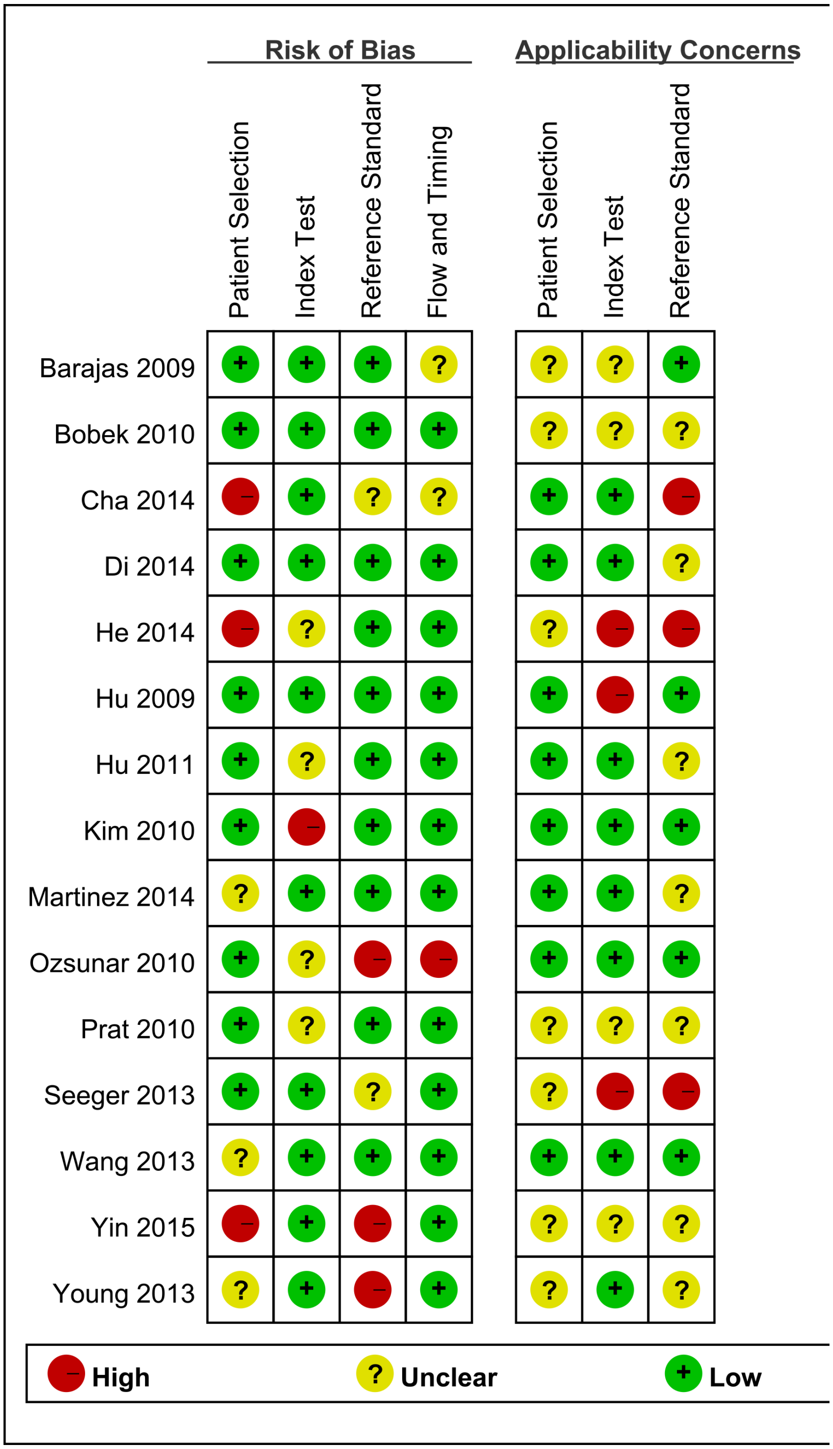

Supplementary Figure 2: Risk of bias and applicability concerns summary: review authors' judgements about each domain for each included study.

Supplementary Table 1: Preferred Reporting Items for Systematic Reviews and Meta-Analyses Protocols (PRISMA-2009 checklist)

See Supplementary File 1
